# Supplementary material for: Development and validation of a questionnaire assessing household work limitations (HOWL-Q) in women with rheumatoid arthritis
Source: PLoS One. 2020 Jul 23;15(7):e0236167. doi: 10.1371/journal.pone.0236167 (PMC7377421; doi:10.1371/journal.pone.0236167)
Supplement: S1 Appendix — (PDF) [file pone.0236167.s001.pdf]

Supplementary Table 1. Consecutive steps/criteria for item´s reduction.

Steps	Criteria applied to each item
1	The inability to discriminate the extreme values of each patient´s selection, according to statistical significance of the t test (p value>0.05)
2	A frequency ≤ 5% in any of the response options.
3	Items correlation calculated (Pearson test) and those items previously identified in steps 2 and 3, with correlations ≥ 0.8 were eliminated.
4	Factor loading < 0.5 (factorial analysis).
5	Items with factor loading in > 1 factor (factorial analysis).
