# Supplementary material for: Development and validation of a questionnaire assessing household work limitations (HOWL-Q) in women with rheumatoid arthritis
Source: PLoS One. 2020 Jul 23;15(7):e0236167. doi: 10.1371/journal.pone.0236167 (PMC7377421; doi:10.1371/journal.pone.0236167)
Supplement: S2 Appendix — (PDF) [file pone.0236167.s002.pdf]

Supplementary table 2. Descriptive statistics for individual items of the HOWL-Q.

Dimension<br>Items	Mean (SD)	Median (P25-P75)	Skewness	Kurtosis
**D1 HOUSEKEEPING**				
5.1_Outdoor sweeping.	0.68 (0.81)	0.50 (0-1)	1.100	0.680
4.1_Mopping.	0.98 (0.89)	1 (0-1)	0.669	-0.247
2.5_Hanging clothes.	0.77 (0.79)	1 (0-1)	0.858	0.285
2.8_Folding clothes.	0.35 (0.60)	0 (0-1)	1.749	3.084
4.2_Changing sheets or making the bed.	0.67 (0.75)	1 (0-1)	0.940	0.440
5.8_Dusting.	0.64 (0.73)	0.50 (0-1)	0.875	0.057
4.12_Washing dishes.	0.59 (0.67)	0 (0-1)	0.973	0.773
2.11_Carry wet clothes.	1 (0.86)	1 (0-2)	0.568	-0.338
4.15_Changing curtains.	1.11 (1.03)	1 (0-2)	0.597	-0.797
2.7_Ironing clothes.	0.93 (0.97)	1 (0-1.25)	0.766	-0.435
3.8_Write.	0.57 (0.70)	0 (0-1)	1.211	1.435
4.9_Cleaning or washing the toilet or bathroom walls.	1.07 (0.93)	1 (0-2)	.0560	-0.526
**D2 INTERACTION WITH OBJECTS AND PERSONS**				
2.2_ Carrying heavy objects.	1.62 (0.96)	1 (1-2)	0.109	-1.055
4.3_ Moving heavy objects or furniture.	1.70 (0.97)	2 (1-3)	-0.032	-1.109
1.2_ Lifting or carrying heavy objects.	1.64 (0.95)	2 (1-2)	0.049	-1.042
3.4_ Pushing the shopping cart.	1.57 (0.99)	1 (1-2)	0.066	-1.063
3.1_ Carrying bags or heavy objects.	1.50 (0.93)	1 (1-2)	0.257	-0.853
2.10_ Carrying water buckets.	1.39 (0.85)	1 (1-2)	0.237	-0.546
6.10_ Carrying or mobilize, some (child, elderly or sick) who is at your charge or care.	1.38 (1.02)	1 (1-2)	0.272	-0.031

2.3_Washing clothes by hand.	1.30 (0.93)	1 (1-2)	0.306	-0.0734
**D3 CARE FOR OTHERS**				
6.6_ Feeding someone (child, elderly or sick) who is in charge or care.	0.53 (0.79)	0 (0-1)	1.573	1.990
6.9_ Supporting to carry out tasks, to some person (child, elderly or sick) who is in charge or care.	0.67 (0.81)	0 (0-1)	1.220	1.045
6.3_ Supporting someone to dress (child, elderly or sick) in charge or care.	0.79 (0.87)	1 (0-1)	0.938	0.159
6.7_ Combing the hair of any person (child, elderly or sick person) who is in charge or care.	0.74 (0.86)	1 (0-1)	1.067	0.472
6.8_ Supporting or accompany in recreational activities or not, any person (child, elderly or sick) who is in charge or care.	0.81 (0.91)	1 (0-1)	1.039	0.319
3.2_ Buying food or grocery shopping.	0.70 (0.86)	0 (0-1)	1.141	0.582
**D4 PERSONAL MOTION AND TRANSPORTATION**				
8.1_ Getting on or off (bus, subway, bike, taxi).	0.90 (0.76)	1 (0-1)	0.637	0.230
1.5_ Standing.	0.72 (0.67)	1 (0-1)	0.412	-0.805
4.14_ Climbing stairs or onto benches.	0.98 (0.80)	1 (0-2)	0.381	-0.562
8.4_ Sitting down or getting up.	0.73 (0.71)	1 (0-1)	0.589	-0.281
3.3_ Walking somewhere to do some diligence.	0.69 (0.78)	1 (0-1)	0.997	0.487
4.8_ Collecting objects from the floor.	0.78 (0.75)	1 (0-1)	0.819	0.478
8.7_ Standing during public transport.	1.09 (0.93)	1 (0-2)	0.523	-0.591
8.9_ Walking or moving on the bus.	1.02 (0.88)	1 (0-2)	0.597	-0.337
**D5 ACTIVITIES REQUIRING MANUAL DEXTERITY**				

3.5_ Receiving change (coins).	0.50 (0.70)	0 (0-1)	1.272	1.059
1.6_ Open the oven or turn on the stove knobs or similar.	0.43 (0.65)	0 (0-1)	1.640	2.881
1.1_ Peeling, cutting or chopping food.	0.79 (0.74)	1 (0-1)	0.551	-0.403
2.6_ Patching or repairing clothes.	0.73 (0.81)	1 (0-1)	1.024	0.611
8.6_ Opening car doors.	0.53 (0.67)	0	1.175	1.139
1.3_ Opening or closing cans and jar lids.	1.38 (0.82)	1 (1-2)	0.357	-0.367
2.1_ Wringing out clothes.	1.20 (0.81)	1 (1-2)	0.295	-0.384
