# Supplementary material for: Development and validation of a questionnaire assessing household work limitations (HOWL-Q) in women with rheumatoid arthritis
Source: PLoS One. 2020 Jul 23;15(7):e0236167. doi: 10.1371/journal.pone.0236167 (PMC7377421; doi:10.1371/journal.pone.0236167)
Supplement: S3 Appendix — (PDF) [file pone.0236167.s003.pdf]

**Cuestionario de actividades instrumentales de la vida diaria en trabajo doméstico México**

- **Instrucciones:** Las siguientes preguntas se relacionan con **las actividades que usted habitualmente realiza en su hogar y/o como empleada del hogar** (en caso que a usted le paguen por atender otra casa). Ninguna respuesta es “buena” o “mala”.
  Por favor, **maque con una “X”** solo una respuesta, la que mejor describa el **GRADO DE DIFICULTAD DURANTE EL ÚLTIMO MES,** que pudiera ocasionarle hacer o realizar cada una de las siguientes actividades; en el caso de que no aplique alguna de las actividades, considere alguna actividad similar.

| **D1** | **ASEO DEL HOGAR** | **Sin dificultad** | **Con alguna dificultad** | **Con mucha dificultad** | **Incapaz de hacerlo** |
|---|---|---|---|---|---|
| 1 | Barrer. | | | | |
| 2 | Trapear. | | | | |
| 3 | Tender la ropa. | | | | |
| 4 | Doblar la ropa. | | | | |
| 5 | Colocar sabanas o hacer la cama. | | | | |
| 6 | Sacudir objetos. | | | | |
| 7 | Lavar trastes. | | | | |
| 8 | Sacar ropa mojada. | | | | |
| 9 | Cambiar cortinas. | | | | |
| 10 | Planchar. | | | | |
| 11 | Escribir. | | | | |
| 12 | Limpiar baños o paredes. | | | | |
| **D2** | **INTERACCIÓN CON OBJETOS Y PERSONAS** | **Sin dificultad** | **Con alguna dificultad** | **Con mucha dificultad** | **Incapaz de hacerlo** |
| 13 | Cargar objetos pesados. | | | | |
| 14 | Mover objetos o muebles pesados. | | | | |
| 15 | Levantar objetos pesados. | | | | |
| 16 | Empujar objetos o muebles pesados. | | | | |
| 17 | Cargar bolsas pesadas. | | | | |
| 18 | Cargar botes o cubetas de agua. | | | | |
| 19 | Cargar o movilizar, a alguna persona (niño, adulto mayor o enfermo) que esté a su cargo o cuidado. | | | | |
| 20 | Lavar ropa a mano. | | | | |
| **D3** | **CUIDADO DE OTROS** | **Sin dificultad** | **Con alguna dificultad** | **Con mucha dificultad** | **Incapaz de hacerlo** |
| 21 | Alimentar a alguna persona (niño, adulto mayor o enfermo) que esté a su cargo o cuidado. | | | | |
| 22 | Ayudar a realizar tareas, a alguna persona (niño, adulto mayor o enfermo) que esté a su cargo o cuidado. | | | | |
| 23 | Vestir a alguna persona (niño, adulto mayor o enfermo) que esté a su cargo o cuidado. | | | | |
| 24 | Peinar a alguna persona (niño, adulto mayor o enfermo) que esté a su cargo o cuidado. | | | | |
| 25 | Ayudar o acompañar en actividades (recreativas o no) a alguna persona (niño, adulto mayor o enfermo) que esté a su cargo o cuidado. | | | | |
| 26 | Comprar alimentos o “hacer la despensa”. | | | | |

| D4 | DESPLAZAMIENTO PERSONAL | Sin dificultad | Con alguna dificultad | Con mucha dificultad | Incapaz de hacerlo |
|---|---|---|---|---|---|
| 27 | Subir o bajar de algún medio de transporte. | | | | |
| 28 | Estar de pie. | | | | |
| 29 | Subir escalones o bancos. | | | | |
| 30 | Sentarse o pararse de un asiento. | | | | |
| 31 | Caminar hacia algún lugar para realizar una diligencia. | | | | |
| 32 | Recoger cosas del suelo. | | | | |
| 33 | Ir de pie en algún medio de transporte. | | | | |
| 34 | Desplazarse dentro de cualquier medio de transporte cuando está en movimiento. | | | | |
| **D5** | **ACTIVIDADES MANUALES O DESTREZA** | **Sin dificultad** | **Con alguna dificultad** | **Con mucha dificultad** | **Incapaz de hacerlo** |
| 35 | Recibir cambio. | | | | |
| 36 | Abrir o encender las llaves de la estufa, horno o similar. | | | | |
| 37 | Pelar, cortar o picar alimentos. | | | | |
| 38 | Remendar. | | | | |
| 39 | Abrir la puerta de un automóvil. | | | | |
| 40 | Abrir o cerrar latas o frascos (nuevos o no nuevos). | | | | |
| 41 | Exprimir. | | | | |
