# Supplementary material for: Development and validation of a questionnaire assessing household work limitations (HOWL-Q) in women with rheumatoid arthritis
Source: PLoS One. 2020 Jul 23;15(7):e0236167. doi: 10.1371/journal.pone.0236167 (PMC7377421; doi:10.1371/journal.pone.0236167)
Supplement: Attachment — Submitted filename: Response to reviewers.docx [file pone.0236167.s004.docx]

**“Development and validation of a questionnaire assessing household work limitations (HOWL-Q) in women with rheumatoid arthritis” (PONE-D-20-04057).**

**RESPONSE TO REVIEWERS AND JOURNAL REQUIREMENTS**

- **Reviewer #1**

**The submitted manuscript titled “Development and validation of a questionnaire assessing household work limitations (HOWL-Q) in women with rheumatoid arthritis” aims to investigate the psychometric properties of a new self-report instrument designed to assess household work limitations in women with rheumatoid arthritis.**

**The topic is of interest, the study is methodologically sound with potential good empirical contribution.**

*Response: We appreciate the reviewer´s comments.*

**Here are some aspects that should be addressed:**

**1.- The flow of the Introduction should be improved. For example, lines 87-92 could be moved after line 64.**

*Response:* We propose and updated flow for the introduction that includes the reviewer´s suggestion.

**2.- Inclusion/Exclusion criteria were not available.**

*Response: Inclusion/exclusion criteria had been added to the Methods section.*

***3.-* As measures of reliability, other statistics should be used (i.e., McDonald’s omega).**

*Response: We have adopted the reviewer´s suggestion and updated the statistical analysis, the results section and reference 32. Thank you for the proposal!*

**4.- Please provide the frequency distribution of the responses to the HOWL-Q individual items using skewness and kurtosis.**

*Response: S2 Appendix has been added to the manuscript with the require information, (Results section).*

**5.- Please specify under which conditions the participants completed the questionnaires in study.**

*Response: We have added a paragraph in the Procedures section.*

**6.- In the data analysis, please provide more details regarding psychometric properties (i.e., intern consistency, construct validity) and a better description of the measures used to test convergent validity.**

*Response: Internal consistency and construct validity are provided; a description of the questionnaires/index used to test convergent validity is provided in table 1.*

- **Reviewer #2**

**The authors proposed a very interesting questionnaire, evaluating household work limitations in rheumatoid arthritis patients. This is an important topic, not considered in the other work addressed questionnaire. Thus, it could provide another aspect of work ability impairment in RA women.**

**The paper is well written and robust from a statistical point of view.**

*Response: We appreciate the reviewer´s comments.*

- **Journal Requirements:**

**When submitting your revision, we need you to address these additional requirements.**

**1. Please ensure that your manuscript meets PLOS ONE's style requirements, including those for file naming. The PLOS ONE style templates can be found at** [**http://www.journals.plos.org/plosone/s/file?id=wjVg/PLOSOne_formatting_sample_main_body.pdf**](http://www.journals.plos.org/plosone/s/file?id=wjVg/PLOSOne_formatting_sample_main_body.pdf) **and http://www.journals.plos.org/plosone/s/file?id=ba62/PLOSOne_formatting_sample_title_authors_affiliations.pdf**

*Response: The PLOS ONE’s style requirements from the manuscript has been reviewed.*

**2. Please amend your current ethics statement to address the following concerns: Please explain why written consent was not obtained, how you recorded/documented participant consent, and if the ethics committees/IRBs approved this consent procedure.**

*Response: The ethic statement in the manuscript has been amended. All the participants gave written informed consent. Patients and controls from sample 1 were surveyed to define and rate household tasks/activities; in them, verbal informed consent was approved by local IRB.*

*(Lertsithichai P. Waiver of consent in clinical observational research. J Med Assoc Thai. 2005;88:275–281)*

**3. We note you have included a table to which you do not refer in the text of your manuscript. Please ensure that you refer to Table 6 in your text; if accepted, production will need this reference to link the reader to the Table.**

*Response: We have confirmed that all the tables included* *have been referred in the text of the manuscript.*
